# Endothelial-like malignant glioma cells in dynamic three dimensional culture identifies a role for VEGF and FGFR in a tumor-derived angiogenic response

**DOI:** 10.18632/oncotarget.4339

**Published:** 2015-06-02

**Authors:** Stuart J. Smith, Jennifer H. Ward, Christopher Tan, Richard G. Grundy, Ruman Rahman

**Affiliations:** ^1^ Children's Brain Tumor Research Centre, School of Medicine, University of Nottingham, Nottingham, UK

**Keywords:** vasculogenic mimicry, rotary cell culture system, angiogenesis, glioma, tumor-derived endothelium

## Abstract

Aims: Recent studies have observed that cells from high-grade glial tumors are capable of assuming an endothelial phenotype and genotype, a process termed ‘vasculogenic mimicry’ (VM). Here we model and manipulate VM in dynamic 3-dimensional (3D) glioma cultures. Methods: The Rotary Cell Culture System (RCCS) was used to derive large macroscopic glioma aggregates, which were sectioned for immunohistochemistry and RNA extracted prior to angiogenic array-PCR. Results: A 3D cell culture induced microenvironment (containing only glial cells) is sufficient to promote expression of the endothelial markers CD105, CD31 and vWF in a proportion of glioma aggregates *in vitro*. Many pro-angiogenic genes were upregulated in glioma aggregates and in primary explants and glioma cells were capable of forming tubular-like 3D structures under endothelial-promoting conditions. Competitive inhibition of either vascular endothelial growth factor or fibroblast growth factor receptor was sufficient to impair VM and downregulate the tumor-derived angiogenic response, whilst impairing tumor cell derived tubule formation. Glioma xenografts using the same cells reveal tumor-derived vessel-like structures near necrotic areas, consistent with widespread tumor-derived endothelial expression in primary glioma tissue. Conclusions: Our findings support studies indicating that tumor-derived endothelial cells arise in gliomas and describe a dynamic 3D culture as a *bona fide* model to interrogate the molecular basis of this phenomenon *in vitro*. Resistance to current anti-angiogenic therapies and the contribution of tumor derived endothelial cells to such resistance are amenable to study using the RCCS.

## INTRODUCTION

The rationale for current anti-angiogenic strategies in high-grade glioma is based upon the targeting of endothelial cells that are recruited from surrounding vascular networks or induced via sprouting of pre-existing vessels. Vascular endothelial growth factor (VEGF), a canonical master regulator of angiogenesis, has been regarded as a common effector of several angiogenic pathways and the anti-VEGF monoclonal antibody bevacizumab (Avastin) is one of few agents to progress to phase III trials. However, whilst the phase II clinical results of VEGF blockade in patients were promising [1, 2], the outcomes of phase III trials have been disappointing with no benefit to overall survival [3, 4]. The assumption of a genetically stable endothelial phenotype in GBM has recently been challenged with evidence of tumor-derived endothelial cells (TDEC), which differ from but can integrate with recruited endothelial cells [5-7]. TDEC are genetically dysregulated cells, which derive from a process intrinsic to the tumor cell termed ‘vasculogenic mimicry’ (VM) [8]. VM was initially described in melanoma but has been observed in leukemia and several solid neoplasms including prostate cancer and glioblastoma (GBM) [9-11]. VM-derived vessels constitute extracellular matrix (ECM)-rich networks lined by tumor cells. Xenografts of human GBM cell lines in rodents were demonstrated to develop blood vessels with human specific endothelial epitopes, harboring tumor specific molecular changes such as epidermal growth factor receptor (EGFR) amplification. Further investigation of VM and the processes that control this cellular behavior [12] will be greatly aided by an ability to simulate this process *in vitro* without the need for an artificial ECM substrate, which will lead to a more comprehensive understanding of the environmental conditions and molecular pathways that promote the adoption of this strategy by cancer cells.

There is a growing recognition conceptually that three-dimensional (3D) culture technologies may more accurately reflect tumor pathophysiology and recapitulate the *in vivo* tumor microenvironment *in vitro*, particularly for complex processes such as VM. One method of generating 3D aggregates in a non-turbulent environment where cells secrete endogenous ECM and growth factors is the Rotary Cell Culture System (RCCS^™^), originally developed by the National Aeronautics and Space Administration (NASA) to study the effects of microgravity on cells and tissues [13]. We have previously demonstrated that the RCCS can recapitulate brain tumor molecular heterogeneity and the hypoxic gradient of primary brain tumors [14]. Here we investigate whether the VM process and the glioma-derived angiogenic response can be characterized and manipulated in the RCCS, thus presenting a model for *in vitro* pharmacological studies aimed at disrupting this potentially important component of tumor growth.

## RESULTS

### High-grade glioma (HGG) and ependymoma cells express endothelial markers in dynamic 3D culture

**Figure 1:**
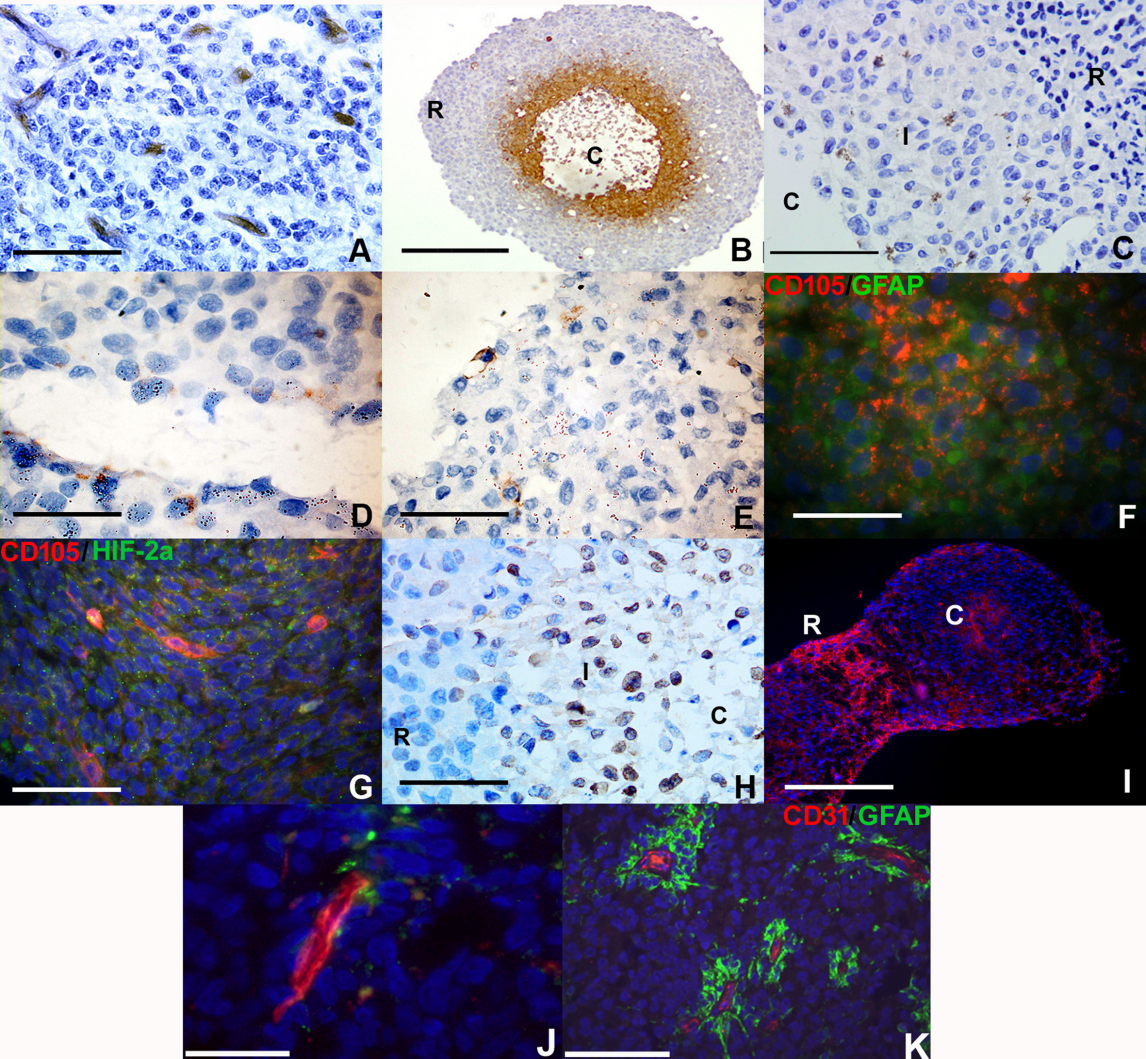
Vascular gene expression in putative brain tumor-derived endothelial cells within RCCS aggregates *in vitro* *Immunological detection of CD105, CD31 and vWF endothelial markers in high grade glioma 3D aggregates.* **A.** Distinct CD105 expression (brown) in microvessel structures in primary high-grade glioma explant tissue. **B.** Hypoxic regions surrounding the necrotic core (*denoted C*) of U87 aggregates are identified using Hypoxyprobe™ (brown); hypoxic staining is absent in the densely cellular peripheral rim (*denoted R*). **C.** GB-1 cells derived from primary high-grade glioma with membranous CD105 expression (brown) at the interface (*denoted I*) between densely cellular aggregate rim (*denoted R*) and necrotic core (*denoted C)*. **D.** KNS42 cells with membranous CD105 expression (brown). **E.** U87 cells with membranous CD105 expression (brown). **F.** Co-localization of CD105 (red) with GFAP (green) in KNS42 cells. **G.** U87 cells exhibit co-localization of CD105 (red) and HIF-2α (green) in aggregate cores and in localized regions. **H.** Widespread vWF expression at the interface (*denoted I*) between densely cellular aggregate rim (*denoted R*) and relatively sparser core (*denoted C)* of KNS42 cells. **I.** Distinct CD105 expression in aggregate core (*denoted C*) and rim (*denoted R*) regions in Res196 ependymoma aggregates. Two aggregates shown that have been harvested and subsequently sectioned in close proximity to each other. **J.** U87 cells with distinct CD31 expression (red) at the aggregate rim / core interface. **K.** Prominent CD31 expression (red) in canonical vessels surrounded by GFAP positive tumour cells (green) in primary HGG tissue. *Scale bar A, C, E-H, J-K = 25μm; D = 10μm; B and I = 200μm*. *All aggregates were cultured for 7-10 days within the RCCS*.

We hypothesized that RCCS culture would induce endothelial-like protein expression in glioma cells in the absence of any endothelial cells or endothelial-promoting media. Primary HGG explant tissue exhibited distinct expression of the immature endothelial marker CD105 (endoglin) (Figure 1A) in blood vessel structures within 7-10 days growth within the RCCS. We have shown here and previously [14] that macroscopic RCCS aggregates (∼0.5 cm – 0.75 cm diameter) can generate intrinsic hypoxic gradients whereby hypoxic glioma cells with low proliferative activity arise in peri-necrotic regions surrounding the aggregate core (Figure 1B). Cells at the interface between the hypoxic core and viable proliferative rim of aggregates expressed the endothelial cell membranous marker CD105 in RCCS 3D tumor-only cultures of the primary cell line GB-1 and in KNS42 and U87 cell line-derived HGG aggregates (Figure 1C–1E).

A marked proportion of CD105 positive cells were also co-positive for GFAP (Figure 1F). Co-staining of CD105 / HIF-2α (a marker of hypoxic adaptation) demonstrated co-localization in the core region (hypoxic and necrotic area) of U87 aggregates (Figure 1G and Supplementary Figure 1A for wide-field view of whole RCCS aggregate). KNS42 RCCS aggregates additionally exhibited high levels of the endothelial marker vWF, with positive cells typically located at the rim / core interface regions (Figure 1H).

Tumor-derived endothelial marker expression was not restricted to GBM cells as aggregates of the primary ependymoma cell line Res196 also exhibited CD105 expression (Figure 1I). Neither CD105 nor CD31 was expressed in KNS42 or U87 2D monolayer cells after 72 hours culture (Supplementary Figure 2A–2D). Prominent expression of the mature endothelial marker CD31 was observed surrounding tubular-like structures in U87 aggregates (Figure 1J and Supplementary Figure 1B for wide-field view of whole RCCS aggregate), which were yet distinct from canonical vessels surrounded by GFAP positive tumour cells in primary HGG tissue. Collectively these findings indicate that a 3D-induced tumour microenvironment is sufficient to promote expression of the endothelial markers CD105, CD31 and vWF in a proportion of high grade glioma and ependymoma cells that reside in distinct tumour aggregate regions.

### Tumor-derived angiogenic response in the RCCS better recapitulates primary glioma angiogenic expression profiles

**Figure 2:**
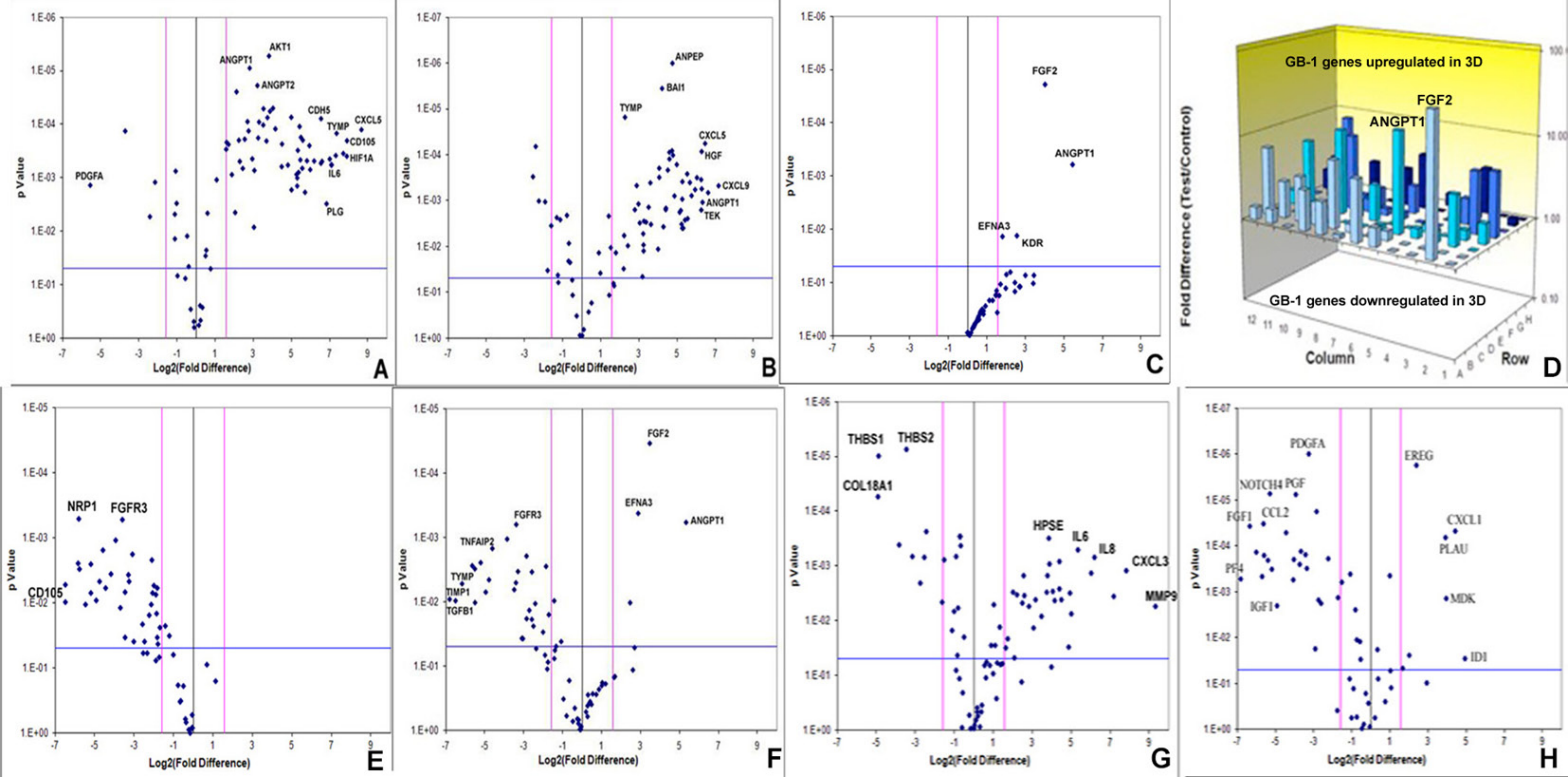
Brain tumor-derived angiogenic expression is upregulated in RCCS culture *in vitro* Volcano plots of angiogenesis array quantitative RT-PCR with a log2 fold difference on x-axis and *p*-value on y-axis. Horizontal blue line represents a *p*-value of 0.05 and vertical pink lines represent a fold change of +/− three. **A.–C.** Angiogenic gene expression between 2D and 3D glioma cultures of KNS42, U87 and GB-1 respectively. **D.** Box plot representation of volcano plot from **C.**, comparing gene expression of GB-1 3D versus 2D expression, illustrating significant upregulation of FGF2 and ANGPT1 genes in 3D culture. Each bar represents one of 84 angiogenic genes. **E.–F.** Angiogenic gene expression in GB-1 2D and 3D cultures respectively, relative to the HGG primary tumor from which the cell line was derived. **G.** Differential angiogenic gene expression in primary GBM explant tissue relative to the 2D monolayer line T7 / 11 derived from this explant. **H.** Differential angiogenic expression between KNS42 / HBMEC heterogeneous aggregates and KNS42 tumor-only aggregates. For all experiments, each dot represents the mean gene expression of one gene assayed in three independent RNA samples from three different 3D aggregates, with selected genes of interest identified on plots. Each experimental condition (e.g. 3D or 2D), is presented relative to each other.

Array quantitative RT-PCR performed for 84 angiogenesis-related genes revealed that KNS42 and U87 GBM RCCS aggregates exhibited significant upregulation of many angiogenic genes relative to corresponding monolayer cultures including FGF1, VEGFR1, VEGFC, ANGPT1, CD31, HGF, CXCL9, TGFβR1 and IGF1 (*p* < 0.001), whilst THBS1, an anti-angiogenic mediator was downregulated (*p* < 0.001) (Figure 2A–2B and Supplementary Tables 1 and 2). GB-1 HGG aggregates showed significant upregulation of four genes including FGF2 and ANGPT1 (*p* < 0.001), though many more genes were upregulated but did not achieve statistical significance (Figure 2C–2D). To determine the resemblance to angiogenic response in primary tumors, GB-1 RCCS aggregates and monolayer cultures were analyzed in comparison to the primary grade III glioma from which the cells were derived. All genes were significantly down-regulated in 2D culture relative to the parental tumor, with the most differentially expressed markers including CD105, NRP1, FGFR3 and MMP2 (*p* < 0.005) (Figure 2E). Although many genes were downregulated in GB-1 3D culture relative to the parental tumor (e.g. FGFR3, TGFβ1 and TYMP), certain genes were now upregulated (e.g. FGF2, ANGPT1 and EFNA3). Additional genes were upregulated but did not achieve significance and the overall pattern of angiogenic-related expression was more similar between 3D culture and parental tumor than between 2D culture and tumor (Figure 2F). Angiogenic gene expression was further compared between an adult GBM (T7/11) acquired directly from surgery and cultured as explant tissue in the RCCS and a monolayer culture derived from this tumor (passage 6). Significant upregulation of many angiogenic genes including MMP9, CXCL3, IL6 and IL8 (*p* < 0.005) and significant downregulation of the anti-angiogenic genes THBS1 and THBS2 (*p* < 0.001) was observed in the explant compared to the monolayer, providing direct evidence that loss of 3D structure was associated with a decreased angiogenic response in monolayers (Figure 2G and Supplementary Table 3). To establish any influence from brain endothelial cell signaling, angiogenic expression in KNS42 / HBMEC co-cultured tumor / endothelial aggregates were compared to tumor-only cultures. Many genes were downregulated in the co-culture including VEGFR1, NOTCH4, FGF1, FGF2, TGFβR1 and MMPs (*p* < 0.001), with a few genes upregulated significantly including CXCL1 and ID1 (Figure 2H and Supplementary Table 4), indicating an overall dampening of the glioma-derived angiogenic response in the presence of canonical brain endothelial cells.

### Tumor-derived vascular markers are expressed *in vivo* and glioma patient tissue

**Figure 3:**
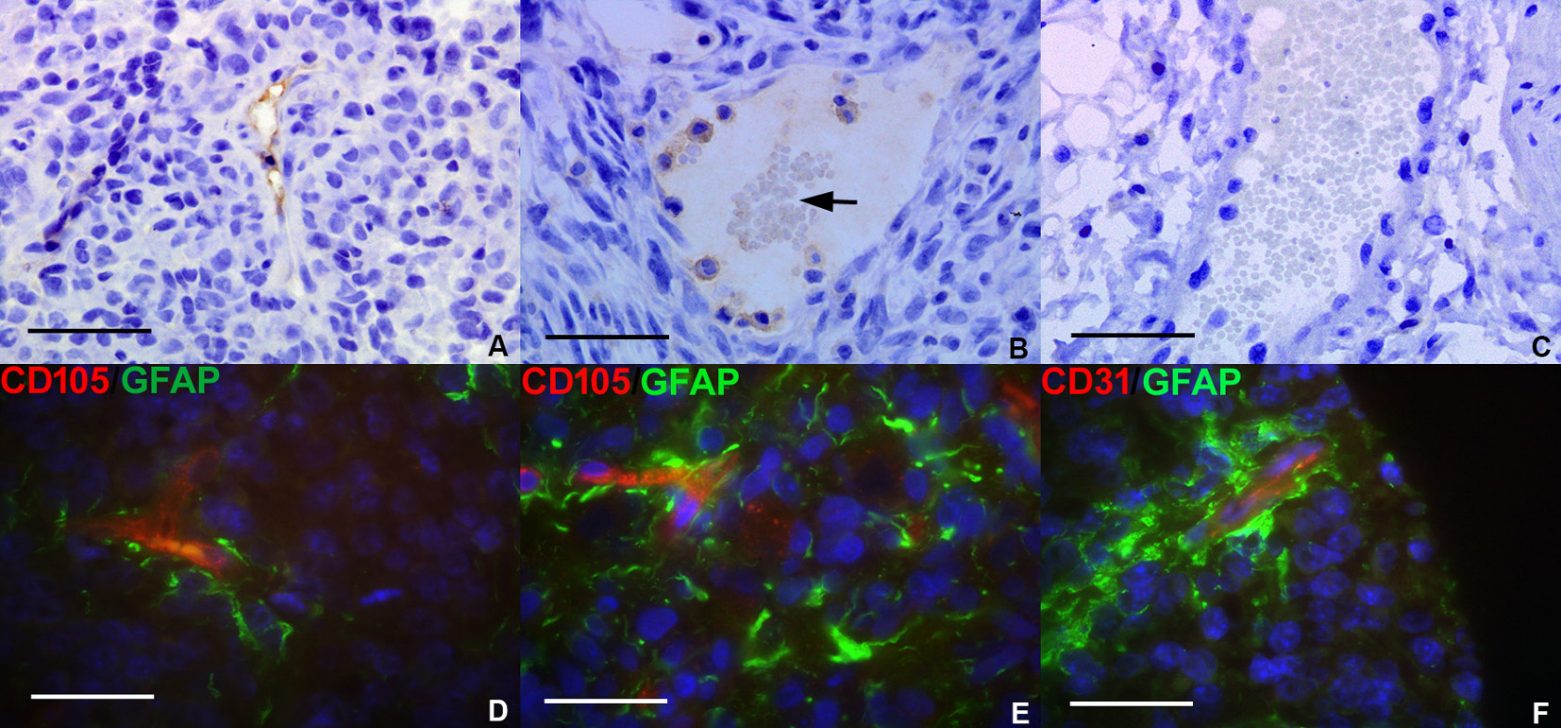
Glioma tumor-derived endothelial marker expression *in vivo* *Subcutaneous human U87 flank xenografts from immunodeficient mice were assessed for tumor-derived endothelial marker expression.* **A.** CD31 human epitope expression (brown stain) lining the wall of a vessel-like structure within the U87 xenograft. **B.** CD105 expression (brown stain) lining vessel-like structures within a xenografted tumor, containing visible erythrocytes (arrow). **C.** Mouse blood vessel from periphery of tumor graft containing erythrocytes but with no positive staining for human-specific CD105. *Immunofluorescence was conducted for tumor and endothelial markers in a selection of pediatric HGG.* **D.–E.** Merged image showing co-localization of CD105 (red) and GFAP (green) on putative TDEC in primary HGG tissue. **F.** Co-localization of CD31 (red) and GFAP (green) in primary HGG tissue. *Scale bar A-C = 25μm; D-F = 20μm*.

To confirm that VM was a phenomenon found in tumor development *in vivo*, U87 GBM cells (at identical passage to that of U87 RCCS culture) were cultured subcutaneously as flank xenografts in nude mice. Within the xenografted tumor mass, blood vessels were positive for CD31 and CD105 protein expression using antibodies specific for the human CD31 and CD105 epitopes (Figure 3A–3B respectively). Erythrocytes were observed within such CD105 positive vessel-like structures, indicating that they were connected to and forming part of the functional mouse vasculature. In contrast, vessels at the periphery of the grafts in the peri-tumoural area were negative for both CD31 and CD105 human protein expression (Figure 3C).

To evaluate the contribution of VM to the peri-vascular niche in human tumors, the co-expression of glial and endothelial markers was investigated in a cohort of primary pediatric HGG patients. A minority population of cells stained co-positive for the astrocytic marker GFAP and endothelial marker CD105 or CD31 suggesting that these may be TDEC within the tumor mass. Co-staining for GFAP / CD105 (Figure 3D–3E) was more frequent than co-staining for GFAP / CD31 (Figure 3F) with CD105 positive TDEC observed in the vicinity of peri-necrotic areas within the tumor.

### Glioma-derived endothelial marker expression may recapitulate an embryonic-like process *in vitro*

**Figure 4:**
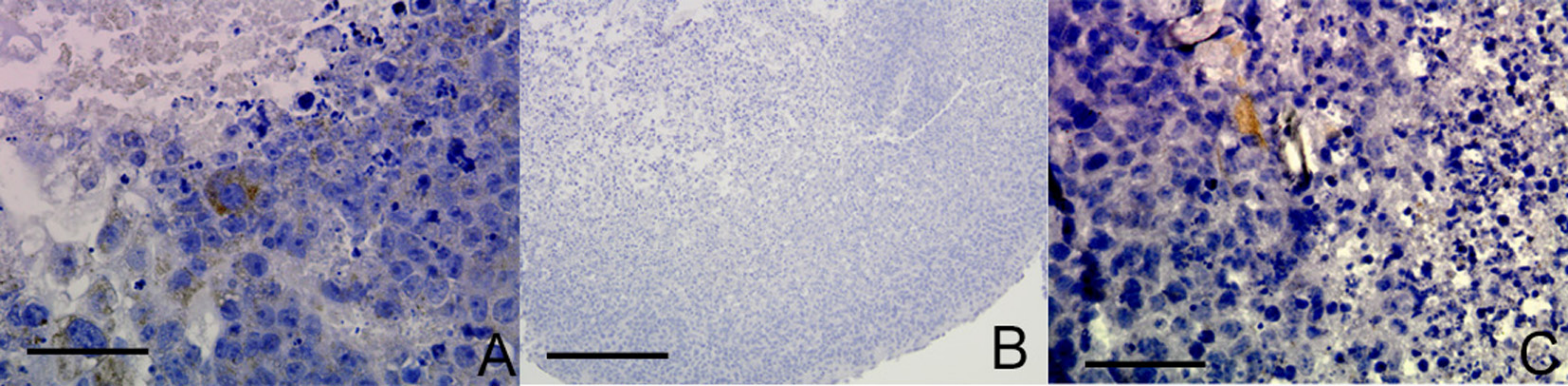
OCT4 overexpression in mouse NSC RCCS aggregates leads to CD105 expression **A.** CD105 expression in mouse ESC (brown stain) **B.** CD105 expression is absent in mouse NSC. **C.** Sparse CD105 expression in mouse NSC with OCT4 overexpression (brown stain). Three independent aggregates for each experiment were fixed and sectioned, with representative images shown. *Scale bar = 25μm*.

We sought to examine whether non-neoplastic embryonic stem cells (ESC) or lineage defined neural stem cells (NSC) also had the capacity under appropriate cell culture conditions to exhibit endothelial-like characteristics. A proportion of glioma cells exhibit stem-like characteristics and we hypothesized that VM may be a recapitulation of a stem-like behavior lost in non-neoplastic lineage defined cells. CD105 was expressed in a proportion of mouse embryonic stem cells (ESC) cultured as RCCS aggregates (Figure 4A), whereas CD105 was completely absent in neural stem cells (NSC) derived from post-natal mice and cultured in the RCCS (Figure 4B). Neural stem cells express three out of four genes required to form a pluripotent cell [21]; the remaining gene encodes the transcription factor OCT4, which has been recently implicated as a potential oncogene [22]. Interestingly, when OCT4 was constitutively over-expressed in the same NSC line, CD105 expression became evident in sporadic cells within aggregates (Figure 4C). CD31 was absent from all ESC and NSC aggregates (Supplementary Figure 2E–2G).

### Tumor-derived angiogenic expression is downregulated upon VEGF / FGFR inhibition *in vitro*

**Figure 5:**
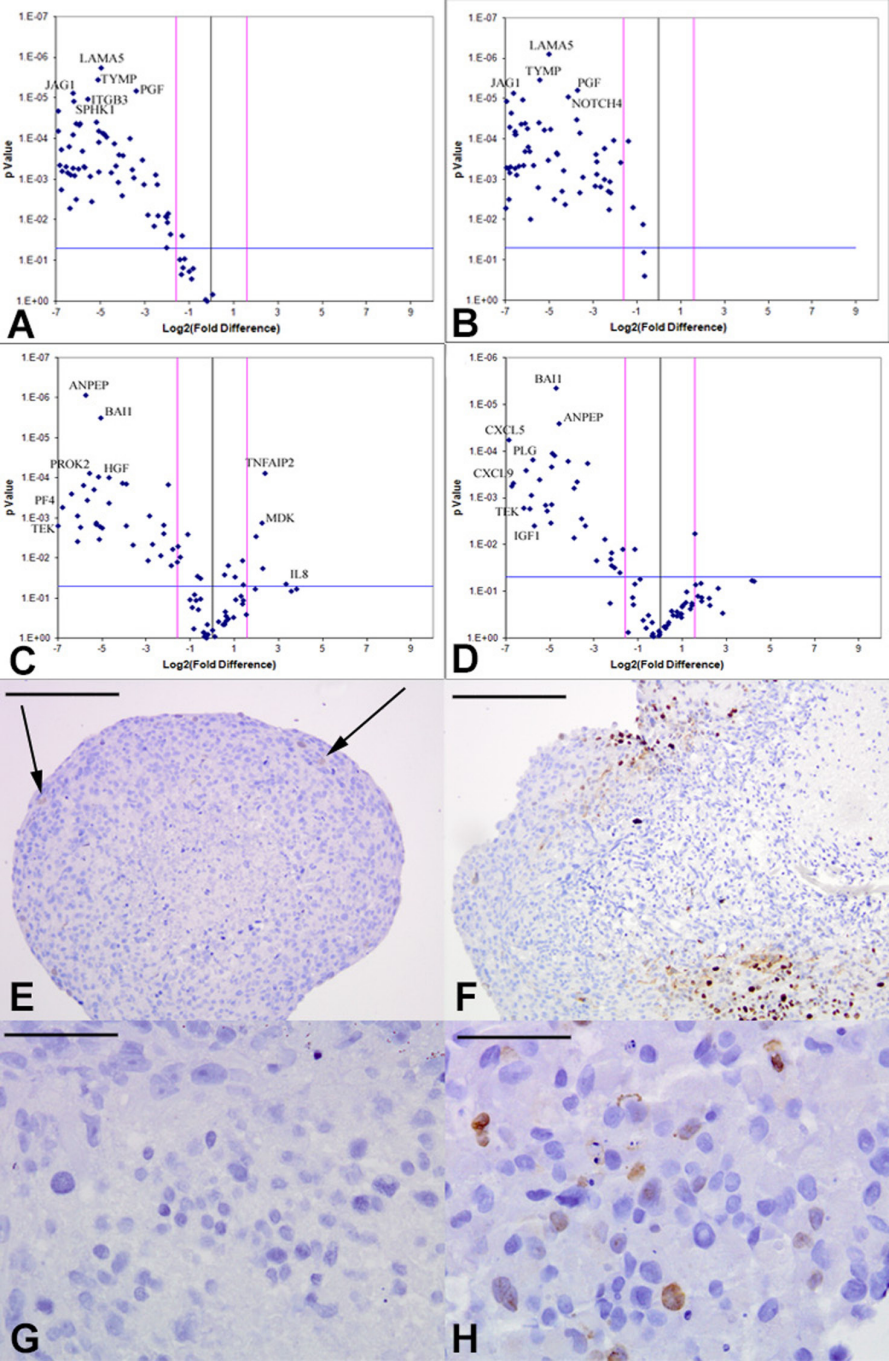
Downregulation of tumor-derived angiogenic response upon VEGF or FGFR inhibition *in vitro* Volcano plots of angiogenesis array quantitative RT-PCR with a log2 fold difference on x-axis and *p*-value on y-axis. Horizontal blue line represents a *p*-value of 0.05 and vertical pink lines represent a fold change of +/− three. Each dot represents the mean gene expression of one gene assayed in three independent RNA samples from three different 3D aggregates, with selected genes identified on plots. **A.–B.** KNS42 aggregates treated with the CBO-P11 VEGF inhibitor or PD16686 FGFR inhibitor respectively, relative to untreated KNS42 aggregates, showing significant downregulation of many angiogenesis-related genes in treated cells. **C.–D.** U87 aggregates treated with CBO-P11 and PD166866 relative to untreated U87 aggregates respectively, showing significant downregulation of many genes in treated aggregates. Angiogenic gene expression depicted represents the mean of three independent experiments each run using triplicate arrays. **E.** Low levels of active proliferation within KNS42 aggregates treated with PD16686, with 4% +/− 0.6 of Ki67 positive cells. **F.** Localized patches of actively proliferating areas within KNS42 aggregates treated with CBO-P11, with 18% +/− 2.3 of Ki67 positive cells. **G.** No Ki67 expression in any cells within U87 aggregates treated with PD16686 (0% +/− 0.0 of Ki67 positive cells). **H.** Sporadic expression of Ki67 within KNS42 aggregates treated with CBO-P11, with 8% +/− 2.9 of Ki67 positive cells. Cells were counted from three independent aggregates and using either a whole aggregate field of view or three different field of views for each aggregate. The mean +/− SEM proportion of Ki67 positive cells relative to total number of cells is given and representative images shown. *Scale bar E-F = 200μm; G-H = 25μm*

Based on our angiogenesis array quantitative RT-PCR data and existing glioma angiogenesis literature [5, 23-25], we selected VEGF and FGFR as candidate mediators of the angiogenic response, observed to be significantly upregulated in the RCCS relative to monolayer cultures (Supplementary Tables 1 and 2) (KNS42: VEGFR2 +135-fold (*p* < 0.001), FGF +6.44-fold (*p* < 0.001); U87: VEGFR1 +22.63-fold (*p* < 0.001), FGF2 +2.85-fold (*p* < 0.01). KNS42 and U87 GBM aggregates were exposed for three days to the VEGF competitive inhibitor CBO-P11 (Calbiochem), which blocks the binding of the VEGF ligand to its receptors VEGFR1 and VEGFR2 or the FGF receptor tyrosine kinase inhibitor PD166866 (Calbiochem), which acts as a competitive inhibitor of the FGF1/2 ligands. We verified the concentrations at which these inhibitors negatively affected cell viability (Supplementary Figure 3) and on this basis selected 10 μM VEGF inhibitor and 15 μM FGFR inhibitor as concentrations at which anti-angiogenic phenotypes (down-regulation of angiogenic genes and disruption of vascular-like structures) could be distinguished without severe compromise to cell viability (Supplementary Figure 4). Many angiogenesis-related genes were downregulated in KNS42 aggregates treated with either inhibitor when compared to untreated aggregates. Prominent amongst downregulated genes in response to VEGF pathway inhibition were TYMP, LEP, HGF, CXCL9, MMP2, VEGFR1 and ANGPT1 (*p* < 0.001) (Figure 5A and Supplementary Table 5). Prominent KNS42 genes significantly downregulated in response to FGFR inhibition were TYMP, HGF, TEK, CXCL9, MMP9, VEGFR1, ANGPT1, FGF1 and FGF2 (*p* < 0.001) (Figure 5B and Supplementary Table 6). KNS42 CD105 and CD31 expression was significantly reduced relative to untreated aggregates upon both drug treatments (*p* < 0.001), though with greater fold change reductions in response to FGFR inhibition (−111.9 for CD31 and −90.7 for CD105) than observed for VEGF inhibition (−30.1 for CD31 and −73.3 for CD105). Two KNS42 genes were significantly downregulated when treatment with both anti-FGFR and anti-VEGF agents was compared (IL6 and CDH5) (Supplementary Tables 5–6). Similarly, many genes were downregulated in U87 as a response to either agent including growth factors and the endothelial specific TEK, a receptor for angiopoietin-1 known to be critical for normal vascular development (Figure 5C–5D). Several genes exhibited reduced expression in a similar manner to that observed for KNS42, e.g. LEP, HGF and TEK (*p* < 0.001) and many U87 cytokines were downregulated in response to either drug, including the pro-angiogenic cytokines CXCL9, CXCL6, CXCL5 and PF4 (CXCL4). Other notable downregulated genes included VEGFR1, TGFβR1, IGF1, EREG (an EGFR ligand), ANGPT1 and HGF (*p* < 0.001) and remodelers of the ECM such as MMP9 (*p* < 0.001) (Figure 5C–5D and Supplementary Tables 7–8). CD31 was significantly downregulated (*p* < 0.001) in response to either agent (−15.08 fold for anti-VEGF and −11.9 fold for anti-FGFR) but CD105 expression was not significantly altered by either drug in U87 aggregates. Notably, the VEGFR1 transcript was significantly downregulated by U87 in response to treatment with either drug (*p* < 0.001) (Supplementary Tables 7–8).

### Vasculogenic mimicry is abrogated upon VEGF/FGFR inhibition *in vitro*

**Figure 6:**
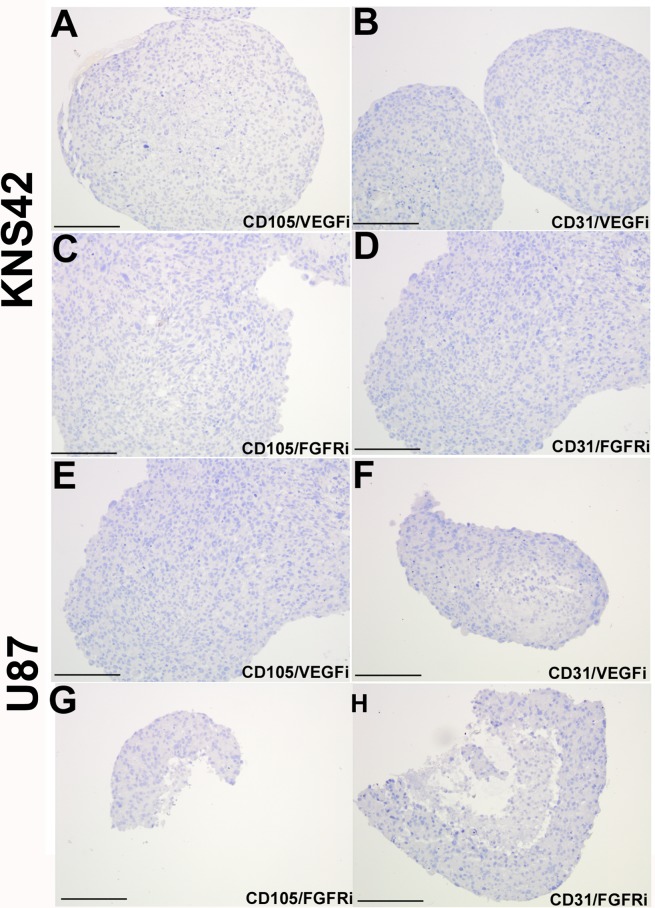
Loss of tumor-derived endothelial marker expression upon VEGF or FGFR inhibition *in vitro* KNS42 and U87 aggregates were cultured for 7 days in the RCCS and subsequently exposed to the CBO-P11 VEGF inhibitor (10 μM) or PD16686 FGFR inhibitor (15 μM) respectively for 3 days. **A.–D.** Absence of CD105 and CD31 staining upon exposure to either inhibitor in KNS42 cells. **E.–H.** Absence of CD105 and CD31 staining upon exposure to either inhibitor in U87 cells. *Scale bar A-H = 200μm. Whole field views of aggregates are shown in all cases to indicate complete absence of endothelial marker staining.*

Glioma aggregates treated with either the VEGF or FGFR inhibitor retained a similar morphologic appearance to untreated aggregates, with a pronounced core region of low cellular density relative to a peripheral rim of high cellular density. (Supplementary Figure 4). KNS42 aggregates exhibited low expression of the proliferation marker Ki67 in response to FGFR inhibition (4% +/− 0.6), but retained foci of high Ki67 expression in response to VEGF inhibition (18% +/− 2.3) (Figure 5E–5F). U87 aggregates exhibited an absence of actively proliferating Ki67 cells when FGFR was inhibited (0% +/− 0.0), but retained sporadic proliferating Ki67 cells when VEGF was inhibited (8% +/− 2.9) (Figure 5G–5H). Exposure of KNS42 and U87 aggregates to either inhibitor (at doses observed not to impair cell viability in monolayer culture, see Supplementary Figure 3) resulted in complete loss of tumor-derived CD105 and CD31 expression (Figure 6), when compared to untreated cells (Figure 1D–1E and 1J). Collectively these findings indicate that TDEC observed in RCCS GBM aggregates is at least in part, dependent on VEGF / FGFR signaling. Whereas lack of TDEC may be coupled to proliferation impairment upon VEGF inhibition in KNS42 and U87 RCCS culture, TDEC absence is independent of proliferation impairment upon FGFR inhibition.

### Glioma cells form endothelial-like tubules in a VEGF/FGFR-dependent manner *in vitro*

**Figure 7:**
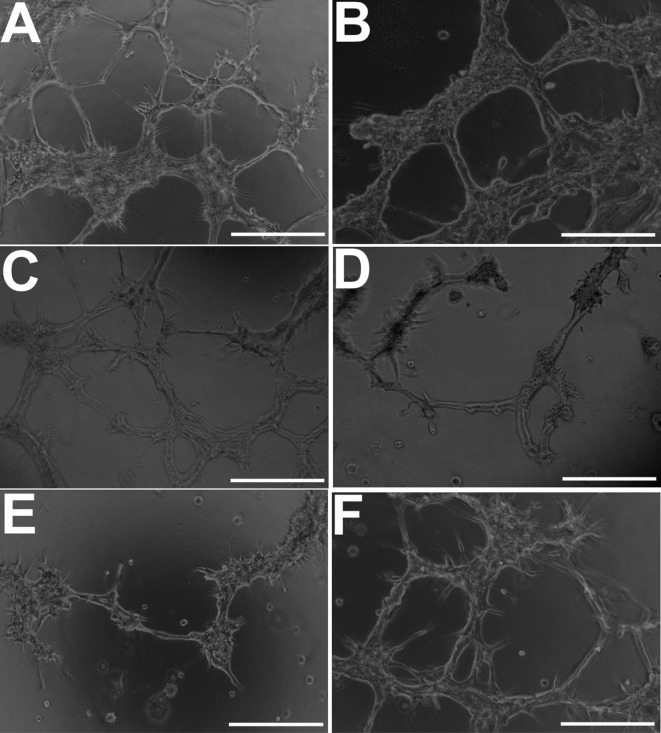
GBM cells develop morphological features of endothelial cells when cultured under endothelial-promoting conditions *in vitro* **A.** Distinct endothelial-like tubular structures comprised of KNS42 cells under normoxia (21% oxygen). **B.** Better structurally-defined tubules when hypoxia (1% hypoxia) is induced using DFO, but not significantly different in mean number (41.8 +/− 3.6) (*p* < 0.05) relative to untreated normoxic cultures (45.4 +/− 6.1). **C.** Moderate impairment of tubular structures upon VEGF inhibition, with significant reduction in mean tubule number (23.4 +/− 4.1 tubules) (*p* < 0.05) relative to untreated cultures. (D-E) Pronounced impairment of closed tubular structures and reduction of tubule number upon FGFR inhibition (9.5 +/− 3.3) (*p* < 0.05) and dual VEGF / FGFR inhibition (4.2 +/− 3.3) (*p* < 0.01). **F.** Exposure to DMSO alone (FGFR inhibitor vehicle) does not impair tubule formation with no significant difference in mean tubule number (47.6 +/− 8.4) (*p* < 0.05) relative to untreated normoxia cultures. *Representative images were taken 48 hours post-seeding and post-exposure to inhibitors. Scale bar = 50μm*.

Under 3D culture conditions (Matrigel) that promote an endothelial phenotype, KNS42 glioma cells (demonstrating an upregulated angiogenic response in RCCS cultures) were capable of generating distinct branching network structures characteristic of endothelial cells (Figure 7A). Branching structures were more pronounced under hypoxic strain, albeit the numbers of tubules were not significantly different (*p* < 0.05) to that observed in normoxic cultures (Figure 7B). VEGF inhibition resulted in mild but significant impairment to tubular structures, with fewer visible tubules relative to normoxic cultures (*p* < 0.05) (Figure 6C), whilst FGFR inhibition or dual VEGF / FGFR inhibition resulted in severe significant impairment of closed tubule formation (*p* < 0.01) (Figure 7D–7E). Exposure to DMSO alone did not lead to a significant change (*p* < 0.05) in branching network formation compared to KNS42 cells under normoxia (Figure 7F).

### Tumor-derived angiogenic expression is a function of the 3D microenvironment *in vitro*

**Figure 8:**
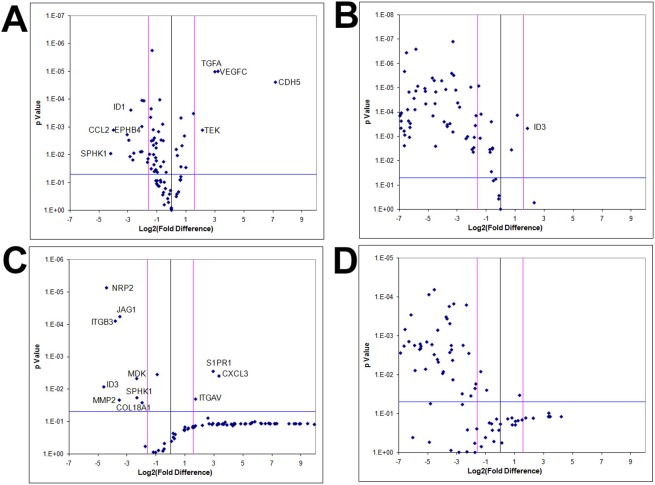
GBM cells exhibit tumor-derived angiogenic expression as a consequence of a 3D microenvironment *in vitro* Volcano plots of angiogenesis array quantitative RT-PCR with a log2 fold difference on x-axis and *p*-value on y-axis. Horizontal blue line represents a *p*-value of 0.05 and vertical pink lines represent a fold change of +/− three. Each dot represents the mean gene expression of one gene assayed in three independent RNA samples from three different 3D aggregates, with selected genes identified on plots. **A.** KNS42 2D monolayers cultured under hypoxia (1% O_2_) relative to KNS42 2D monolayers cultured under normoxia (21% O_2_), showing significant downregulation of several angiogenesis-related genes in hypoxic cells with only CDH5, TGFA, VEGFC and TEK upregulated under hypoxia. **B.** KNS42 2D monolayers cultured under hypoxia (1% O_2)_ relative to KNS42 3D RCCS aggregates, showing significant downregulation of the majority of angiogenesis-related genes in hypoxic monolayers with only ID3 upregulated. **C.** U87 2D monolayers cultured under hypoxia (1% O_2)_ relative to U87 2D monolayers cultured under normoxia (21% O_2_), showing significant downregulation of several angiogenesis-related genes in hypoxic cells with only CXCL3, S1PR1 and ITGAV upregulated under hypoxia. **D.** U87 2D monolayers cultured under hypoxia (1% O_2)_ relative to U87 3D RCCS aggregates, showing significant downregulation of the majority of angiogenesis-related genes in hypoxic monolayers.

To examine whether angiogenic expression is exclusively a hypoxia-driven event independent of the RCCS, KNS42 and U87 monolayer cells were cultured under hypoxic conditions (1% O_2_) and angiogenic gene profiles were compared to RCCS 3D cultures and normoxic (21% O_2_) monolayer cultures. The majority of angiogenic-related genes did not demonstrate significantly upregulated expression in hypoxia monolayer culture relative to normoxic monolayer culture for both KNS42 and U87 cells, whereas several genes were significantly upregulated under normoxia (*p* < 0.001) (Figure 8A and 8C respectively). For both KNS42 and U87 cells, the majority of angiogenic-related genes were significantly upregulated in 3D RCCS culture relative to 2D hypoxic culture (*p* < 0.001) (Figure 8B and 8D respectively). No gene was significantly upregulated in U87 2D hypoxia culture (Figure 8D) and only ID3 was significantly upregulated in KNS42 2D hypoxia culture (*p* < 0.001) (Figure 7B). These findings suggest that hypoxia alone within the RCCS environment per se does not trigger VM and a tumor-derived angiogenic response in GBM cells *in vitro*.

## DISCUSSION

The induced expression of the endothelial markers CD105, CD31 and vWF in HGG cells within the dynamic RCCS was typically observed at the interface between the necrotic core of the aggregate and highly cellular rim. As we have previously showed that this region is hypoxic but viable [14], our finding is consistent with studies indicating that hypoxia is a key microenvironmental cue promoting the microvascular niche. Furthermore, culture conditions promoting endothelial proliferation enhanced the TDEC phenotype in glioma cells with respect to tubular structure formation in Matrigel and which was moderately more distinct under hypoxic strain. However our results indicate that hypoxia alone was insufficient to induce VM-related and tumor-derived angiogenic changes in monolayer cultures, but rather that the key trigger in the context of our experiments was the three-dimensionality of the RCCS and Matrigel cultures. This implies that cellular interaction is an important determinant of tumor-derived VM in our culture systems and which may be potentiated by induced hypoxic strain as observed in the tubule-forming assay. This finding is consistent with a report of hypoxic promotion of VM in a 3D ovarian carcinoma culture model [26].

We demonstrate CD105 / CD31 TDEC co-expression and VM structures in U87 xenografts *in vivo* and CD105 / GFAP and CD31 / GFAP co-localization in primary HGG tissue consistent with previous studies [10], albeit our interpretation is limited to a flank model rather than orthotopic brain. This is evidence against the possibility that TDEC and VM are simply a function unique to the RCCS, but rather support the notion that these are distinct biological processes that may contribute to neo-vascularization within primary HGG tissue. The importance of endothelial-like cells to tumor growth may be best understood in the creation of stimulatory cell-cell contacts in the peri-vascular niche, as opposed to the creation of a functional vasculature. This has previously been suggested by the observation of increased growth of glioma cells co-cultured with endothelial cells. We have previously documented that co-localization of brain tumor and endothelial markers frequently occurs in peri-necrotic regions of primary HGG and that CD105 expression is an indicator of poor prognosis in a cohort of pediatric HGG (*n* = 150) [27]. Our data suggests that the presence of necrotic cells may stimulate tumor cells to acquire a TDEC phenotype. Glioma cell / endothelial cell co-cultured aggregates displayed a greatly altered pattern of gene expression compared to glioma-only aggregates, with many of the genes putatively implicated in VM including NOTCH4, TGFβ, HGF and CD31 being significantly downregulated. One explanation for this may be that in the presence of sufficient pre-existing endothelial cells there is less of a selective pressure for glioma cells to transdifferentiate to TDEC and undergo VM. It is plausible that the transdifferentiation of TDEC under hypoxic conditions is in part due to a lack of endothelial precursors with which to re-constitute the neo-vascular niche. Several angiogenesis-related molecules such as Laminin, Neuropilin-2 and CD105 have been reported to be enriched in glioma stem cells capable of forming vascular structures *in vitro* [8], implicating that TDEC transdifferentiation is facilitated by a glioma stem-like phenotype.

The signaling pathway(s) by which TDEC and VM emerge are still under investigation. We show that endothelial markers are expressed in ESC aggregates and NSC aggregates which overexpress OCT4, but not wild-type NSC. This finding implies that an induced pluripotent-like state is necessary to generate VM in normal lineage-restricted neural cells, whereby OCT4 overexpression is necessary for CD105 expression in NSC. An association between genes linked to an undifferentiated embryonic-like phenotype and VM-positive tissues has been reported in VM studies from aggressive melanoma [28]. Our finding suggests that VM in brain tumor cells recapitulates a process that relies on pluripotent-like features, perhaps similar to an embryonic-like process that originates in the transformed neural precursor cells. The expression of endothelial markers in lineage defined NSC upon forced OCT4 expression suggests a similar process could potentially underlie the ability of cancer cells to undergo the same change, whereby some glioma cells under selection pressure at least transiently revert to a primitive glioma stem cell state, prior to exerting tumor-derived phenotypes.

Several angiogenesis-related genes were upregulated within glioma RCCS cultures in comparison to monolayer cultures, including components of the FGF and TGFβ signaling pathways, suggesting that these factors are mediators of VM that may be targeted therapeutically. Indeed downregulation of TGFβ in GBM U251 cells abrogates the ability to form VM-associated tubular structures on Matrigel with an associated decline in MMP-2 activity [29]. The PI-3 kinase network mediated by MMP-2 upregulation has previously been implicated in melanoma VM [30], consistent with our observation that MMP-2 was one of the most downregulated genes in response to FGFR or VEGF inhibition. Other metalloproteinases, e.g. MMP-9, were among the genes most upregulated by RCCS 3D culture, consistent with a significant association between VM and MMP-9 expression in clinical glioma specimens [31] and in tumor / endothelia co-cultures [32]. It is plausible therefore that VM may be associated with the aggressive and invasive nature of gliomas. The remodeling of ECM has been shown to provide the space needed for VM and is regulated by metalloproteinases [33]. Therefore the upregulation and secretion of endogenous ECM we have previously observed in RCCS aggregates [14], coupled with the upregulation of metalloproteinases shown in this study, suggests that ECM remodeling may precede VM formation and glioma-derived endothelial marker expression.

Our results suggest that small molecule inhibitors that competitively bind to VEGF and FGFR both have an effect in abrogating the development of VM, though FGFR may have a more fundamental role. Approaches such as the monoclonal antibody bevacizumab that target free VEGF ligand may have a limited inhibitory effect on angiogenesis if FGFR dependent VM plays a significant role. Although TDEC failed to express VEGFR in one study, others have reported that VEGFR2 is expressed on these cells [5, 34]. Blockade of VEGF ligand or gene silencing of VEGFR2 appears to inhibit the differentiation of tumor endothelial progenitors into endothelium, but not the differentiation of glioma stem-like cells into immature tumor endothelial progenitors [6]. Similarly, glioma stem-like cells formed a higher number of vascular structures in a tubule-forming assay and preferentially expressed high levels of VEGFR2 (relative to parental GBM cells), which was abrogated upon targeting of VEGFR2 using a monoclonal antibody [35]. This finding suggests that the VEGF-dependent upregulated angiogenic response, endothelial marker expression and vascular structure formation observed in our study, may be due to a VEGF-mediated role in the maturation of TDEC. As VEGF inhibition still resulted in a reduction of endothelial markers, our results also imply that the differentiation of a proportion of glioma cells into TDEC in the RCSS may be VEGF-dependent. However, branching network formation and gene expression of endothelial markers was more strongly abrogated by FGFR inhibition in our study, suggesting that FGF signaling may be functionally more relevant to glioma VM, at least in the context of our model. Studies concerning VM in glioma have not yet addressed the direct clinical significance of this process in the course of tumor initiation, progression and recurrence. Anti-angiogenic therapy trials may be required to consider VM as a possible mechanism of therapeutic failure, as TDEC often do not express the same levels of receptors that *bona fide* endothelial cells display.

The RCCS provides a practical way of modeling VM within a human-specific model *in vitro*, allowing for biological manipulation and investigation of this potentially important mechanism of resistance to anti-angiogenic therapy. To our knowledge this is the first report of TDEC and VM in a 3D glioma culture model. The investigations presented here suggest that FGFR plays a significant role in VM but that VEGF, OCT4 and other pathways also play important roles and that the process is intrinsically linked to cell-to-cell interaction within a 3D context, hypoxia and necrosis. Pharmacological intervention in the RCCS in comparison to rodent orthotopic models will be required to further validate the RCCS (and indeed alternative 3D culture models) as a surrogate to study and therapeutically manipulate VM. Further work will undoubtedly reveal a more complex molecular basis of VM and elucidate the therapeutic potential of targeting VM in malignant tumors.

## MATERIALS AND METHODS

### Monolayer and RCCS cultures

KNS42 (pediatric GBM) [15] and Res196 [16] (pediatric ependymoma) were maintained in Dulbecco's modified Eagle's medium (DMEM) / F12 (Invitrogen); U87 (adult GBM), T7 / 11 (adult primary GBM) and GB-1 (pediatric mixed glial-neuronal grade III tumor, previously described by us) [17] in DMEM (Invitrogen); E14TG2A (mouse ESC) in Glasgow minimal essential medium (Sigma); HBMEC [18] (brain microvascular endothelial cells) in Roswell Park Memorial Institute (RPMI) medium 1640 (Sigma); and post-natal day 9 mouse NSC with Oct4 over-expression in B27 / N2 / DMEM / F12 neurobasal medium. NSC with the Oct4 transgene was generated as described [19]. All media were supplemented with 10% fetal bovine serum (except HBMEC which was supplemented with 20% fetal bovine serum). A 1:1 tumor:endothelial ratio media was used as co-culture media. 3D RCCS (Synthecon, Luxembourg) culture was commenced by introducing 1 × 10^6^ cells as a single cell suspension into a 10 ml rotating vessel containing media as per monolayer cultures. Once a visible aggregate formed (typically by 1-2 days), revolution speed was adjusted to balance the Coriolis force against gravity in order to maintain the aggregate in stationary free-fall. Harvested aggregates (typically after 7-10 days) were either fixed in 4% paraformaldehyde or stored frozen at −80°C until required for further analysis. Exposure to angiogenic inhibitors was achieved by culturing RCCS aggregates in media containing either 10 μM anti-vascular endothelial growth factor (VEGF) inhibitor (Calbiochem, CBO-P11) or 15 μM anti-fibroblast growth factor receptor (FGFR) inhibitor (Calbiochem, PD166866) for three days. KNS42 and U87 hypoxic monolayer culture was achieved via a 24 hour incubation period at 1% oxygen using a Glove Box Workstation (M.Braun, UK). For all subsequent molecular analyses, at least three independent RCCS aggregates were cultured and harvested. To determine the proportion of actively proliferating cells in glioma aggregates upon anti-angiogenic treatment, a Counter-Pen™ Cell Counter (VWR International, UK), was used to count Ki67 positive and Ki67 negative cells from three independent aggregates and using either a whole aggregate field of view or three different field of views for each aggregate. The mean +/− standard error (SEM) percentage of Ki67 positive cells relative to total number of cells was calculated.

### Pediatric high-grade glioma tissue microarray

Pediatric high-grade gliomas (HGG) were surgically collected ante-mortem at UK pediatric neurosurgical centers (between 1987 and 2007) and banked by the Children's Cancer Leukemia Group (UK CCLG) through Nottingham Children's Brain Tumor Research Centre, with diagnosis confirmed by central pathological review. The project has ethics (UK ethics/R&D number PO 059801 and OG 080002) and Biobank approval (11/EM/0076) for collection of fresh tumour samples from surgery.

### Immunological detection

Standard immunohistochemistry procedure [20] was followed with the following antibody dilutions: anti-Ki67 (Dako, clone MiB-1), 1:50; anti-CD31 (Dako, clone JC70A), 1:50; anti-CD105 (Abcam, ab49679), 1:100; anti-vWF, 1:800 (Abcam, ab6994). Standard immunofluorescence was followed with primary antibody dilutions of anti-CD31, anti-CD105 and anti-vWF as per immunohistochemistry; anti-GFAP (Abcam, ab726), 1:1000; anti-HIF-2α (Abcam, ab199) 1:100 and secondary antibody combinations of Alexa488-conjugated goat anti-rabbit, 1:200 and Alexa555-conjugated goat anti-mouse, 1:200. Images were taken using a Nikon ECLIPSE 90i light microscope fitted with a Hamamatsu OCRA-ER camera using three fluorescent light filters. For KNS42 cell cultures undergoing Hypoxyprobe™ (Hypoxyprobe Inc., Burlington, MA) analysis, the Hypoxyprobe™ reagent (pimonidazole) was applied two hours before harvesting at 200 μM concentration. Samples were then fixed as standard and immunohistochemistry performed using the primary antibody supplied with the Hypoxyprobe™ kit at 1:200 concentration. Three independent RCCS aggregates were fixed and sectioned prior to immunohistochemical detection and representative qualitative images taken from five slides.

### Array quantitative reverse transcriptase (RT)-PCR

cDNA was synthesized from 1 μg total RNA using the RT^2^ First Strand Kit (SABiosciences) according to the standard protocol. Array quantitative RT-PCR was performed using the human angiogenesis array (PAHS-024D) (SABiosciences) containing 84 angiogenesis-related genes and according to manufacturer guidelines using 96 well plates. The array was run on a CFX96 (BioRad) instrument for 10 minutes at 95°C followed by 40 cycles of 95°C for 15 seconds and 60°C for one minute. Each cycle was followed by a plate read and CT values were calculated for each well in triplicate with each array experiment conducted on three independent aggregates or three independent monolayer cultures for each line.

### *In vivo* xenografts

Six nude mice were implanted subcutaneously in the flank with 1 × 10^6^ U87 glioma cells. Tumors were harvested after four weeks growth, formalin fixed and paraffin embedded. Immunohistochemistry against human-specific vascular markers was undertaken in a standard fashion. All procedures were in compliance with the Animals (Scientific Procedures) Act 1986 with mice having access to sterile food and water *ad libitum*, environmental enrichment and ample sterile bedding.

### Matrigel tubule formation assay

Endothelial-like tubule formation was assessed using the BioCoat Angiogenesis System (BD Biosciences). Briefly, 2 × 10^4^ KNS42 cells were seeded onto Matrigel in each well of a 24-well plate using endothelial medium EBM-2 (Lonza) containing 10 ng / ml FGF and 25 ng / ml VEGF supplemented with either: VEGF inhibitor; FGFR inhibitor; both VEGF and FGFR inhibitors; 100 μg / ml desferrioxamine (DFO) hypoxic inducer; or DMSO alone. Images of tubules were taken 48 hours post-seeding using a light microscope. Cells were plated in triplicate wells and the experiment repeated three times, with representative qualitative images taken. To semi-quantitate the degree of vessel-like formation, the number of fully-formed structurally intact tubules, deemed as those with closed sides, were counted per well. The mean number of tubules +/− SEM was determined from triplicates of three independent experiments (*n* = 9).

### Biostatistics

Analysis of the array quantitative RT-PCR data was undertaken using the accompanying Excel based statistical package (SABiosciences) and confirmed using SPSS in order to determine significant fold changes. Gene expression data was presented consistently using volcano plots where fold-change was plotted directly against statistical significance. *P*-values were considered significant at less than 0.05 throughout. An independent student *t*-test (SPSS) was used to determine significant differences in vessel-like tubule formation in the Matrigel assay.

## SUPPLEMENTARY MATERIAL FIGURES AND TABLES


