# Supplementary material for: Endothelial-like malignant glioma cells in dynamic three dimensional culture identifies a role for VEGF and FGFR in a tumor-derived angiogenic response
Source: Oncotarget. 2015 Jun 2;6(26):22191–205. doi: 10.18632/oncotarget.4339 (PMC4673156; doi:10.18632/oncotarget.4339)
Supplement: Supplementary file 1 [file oncotarget-06-22191-s001.pdf]

Endothelial-like malignant glioma cells in dynamic three dimensional culture identifies a role for VEGF and FGFR in a tumor-derived angiogenic response

Supplementary Material

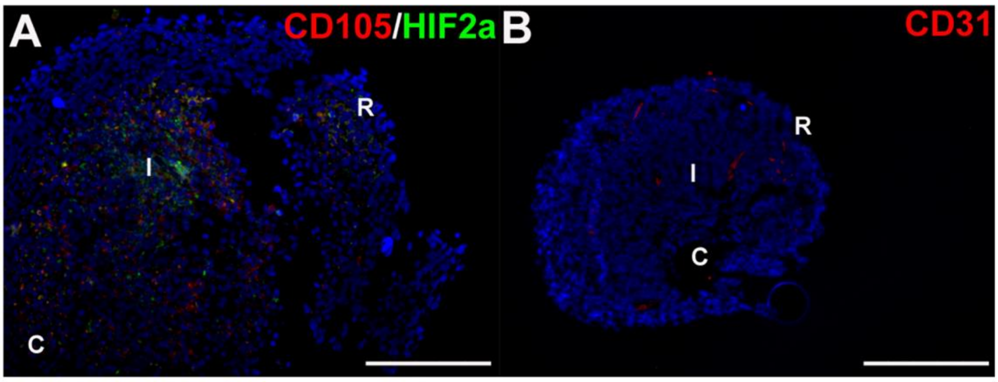

Supplementary Figure 1: Whole-field RCCS U87 aggregate views of CD105 and CD31 positive cells. (A) Location of CD105 positive cells in relation to aggregate location and to HIF-2α positive cells. (B) Location of CD31 positive cells in relation to aggregate location. Aggregate core, rim and interface regions are denoted as *C*, *R* and *I* respectively. *Scale bar = 200µm.*

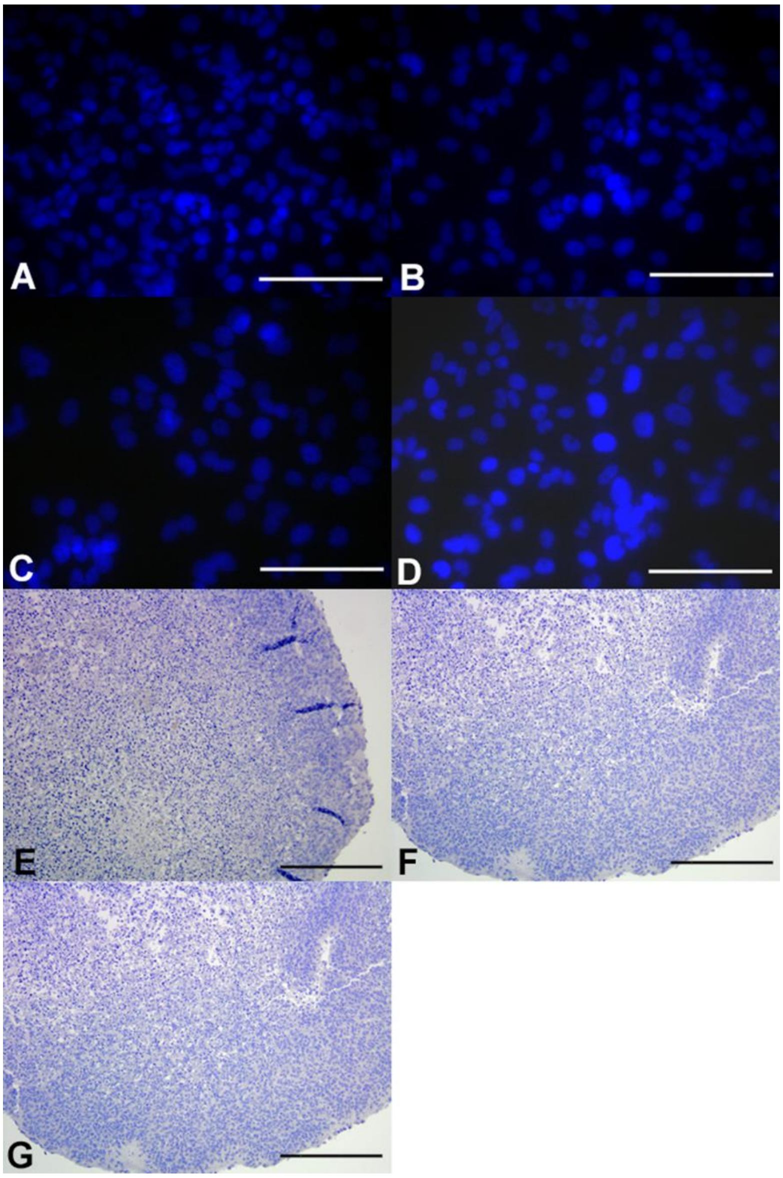

Supplementary Figure 2: Negative staining for CD105 and CD31 in monolayers and stem cell aggregates (CD31). (A) Merged image with no positive (red) staining for CD105 on immunofluorescence (IF) of a KNS42 2D monolayer. (B) Merged image with no positive CD105 staining (red) on IF of a U87 2D monolayer. (C) Merged image with no positive CD31 staining (red) on IF of a KNS42 2D monolayer. (D) Merged image with no positive CD31 staining (red) on IF of a U87 2D monolayer. In *A-D* monolayer cells were cultured for 72 hours prior to staining with either endothelial marker. (E-G) Immunohistochemistry against CD31 on aggregates of ESC (E), NSC (F) and NSC with OCT4 overexpression (G) with no discernible positive staining. *Scale bar A-D = 25µm E-G = 200µm.*

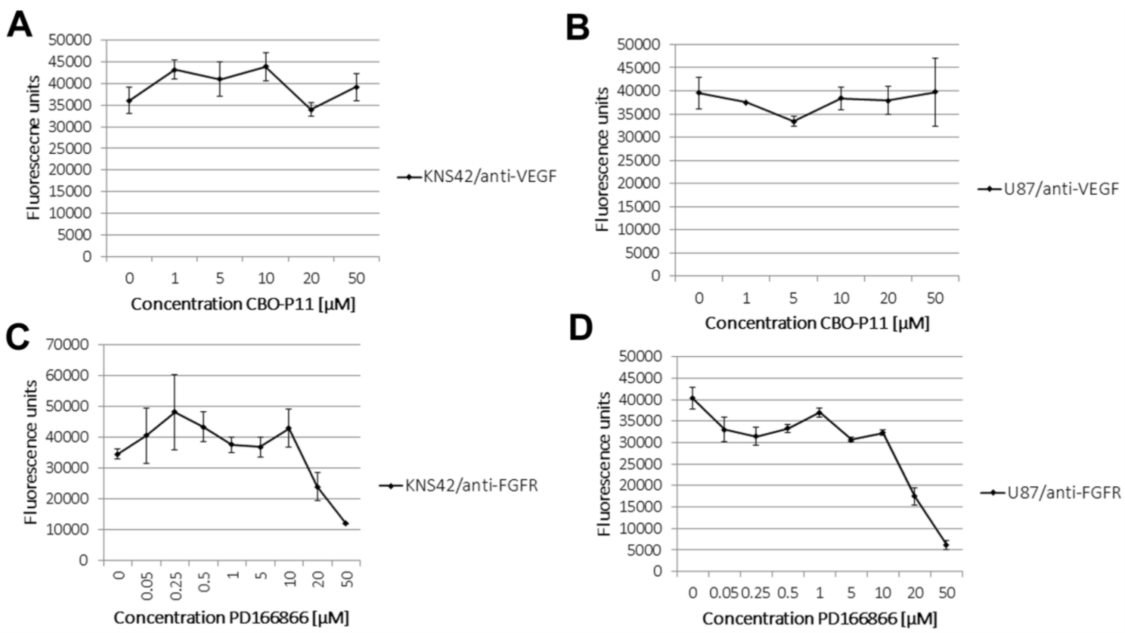

Supplementary Figure 3: KNS42 and U87 RCCS aggregate viability upon exposure to either VEGF inhibitor (CBO-P11) or FGFR inhibitor (PD166866). (A-B) Exposure of either glioma aggregate to up to 50 µM VEGF inhibitor resulted in no impairment of cell viability. (C-D) Exposure of either glioma aggregate to FGFR inhibitor, resulted in marked loss of cell viability at concentrations above 10 µM. Based on this data, 15 µM and 10 µM were chosen as VEGF inhibitor and FGFR inhibitor concentrations respectively, by which to monitor anti-angiogenic response without dramatically impairing cell viability.

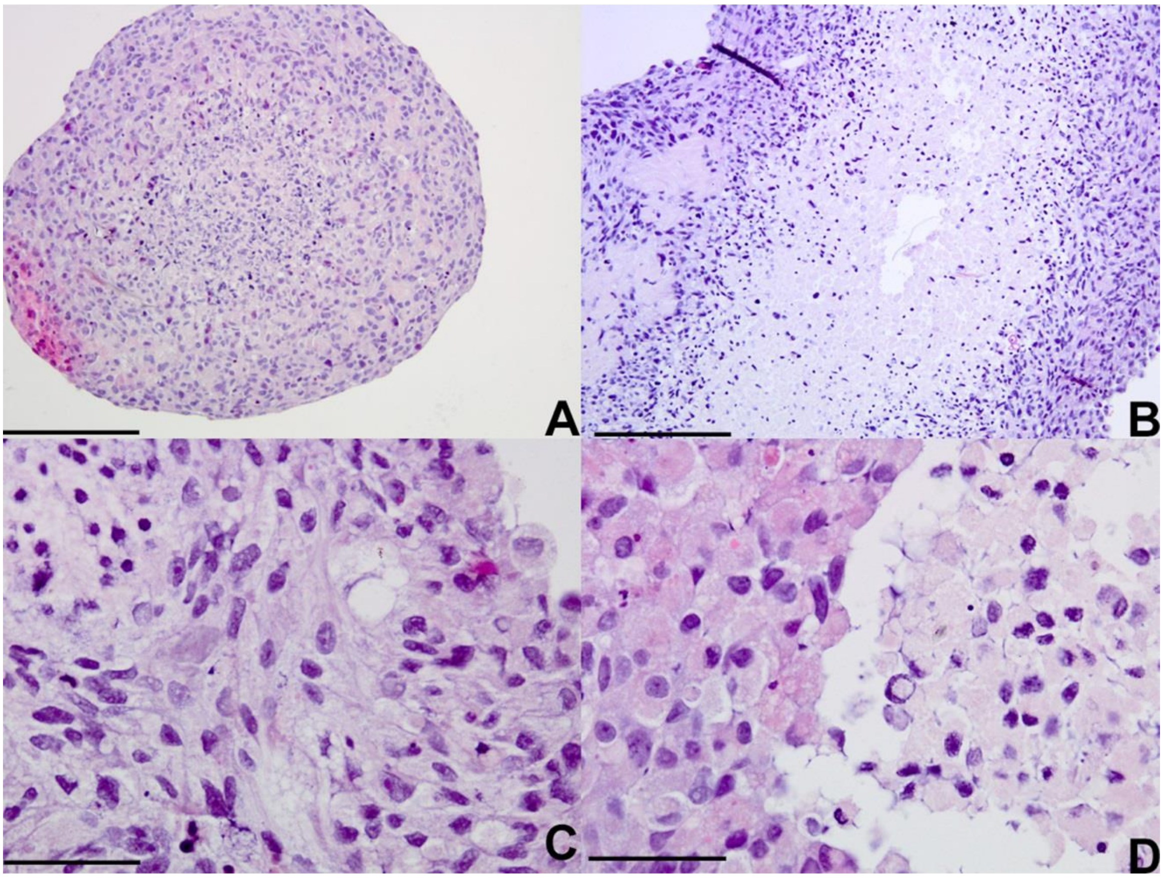

Supplementary Figure 4: Hematoxylin and eosin stained sections of anti-angiogenic drug treated aggregates. (A) KNS42 aggregate treated with 15µM PD166866 (FGFR inhibition) (B) KNS42 aggregate treated with 10µM CBO-P11 (VEGF inhibition) (C) U87 aggregate, FGFR inhibition (D) U87 aggregate, VEGF inhibition. *Scale bar A&B = 200µm C&D = 25µm*.

Supplementary Table 1: Genes significantly differentially expressed between 3D and 2D KNS42 GBM cultures.

Gene	Mean Fold Change 3D to 2D	P value (t-test)
TIMP3	402.64	0.0001
HGF	238.86	0.0002
LEP	236.66	0.0004
IL6	208.42	0.0004
IFNG	163.52	0.0001
PLG	158.68	0.0004
CDH5	135.92	0.0006
VEGFR1	135.92	0.0006
ANGPT1	127.70	0.0004
LECT1	113.51	0.0031
TNF	96.56	0.0005
PTGS1	93.05	0.0001
PF4	92.84	0.0005
CCL11	72.50	0.0005
IFNB1	62.68	0.0007
CXCL9	60.97	0.0003
IFNA1	55.72	0.0005
IGF1	51.98	0.0019

MMP9	47.95	0.0002
CXCL5	47.07	0.0007
CXCL1	44.84	0.0002
THBS2	43.92	0.0005
IL8	42.71	0.0001
CXCL6	41.16	0.0008
PLXDC1	40.04	0.0003
IL1B	39.21	0.0010
MMP2	39.12	0.0014
ENG	38.23	0.0008
TEK	31.85	0.0017
ANGPTL4	31.63	0.0001
CXCL10	27.99	0.0006
BAI1	22.58	0.0002
TGFA	22.32	0.0006
EREG	17.75	0.0001
ANPEP	16.26	0.0000
ANGPTL3	14.59	0.0001
TYMP	14.09	0.0000
FIGF	13.30	0.0001
ANGPT2	12.85	0.0002
TNFAIP2	11.52	0.0001

PROK2	11.42	0.0001
HAND2	9.80	0.0001
CXCL3	9.51	0.0002
VEGFC	9.23	0.0000
SERPINF1	8.28	0.0007
EFNA3	8.15	0.0085
KDR	7.67	0.0004
PGF	6.98	0.0001
STAB1	6.71	0.0001
FGF1	6.44	0.0001
CD31	5.84	0.0002
TGFBR1	5.44	0.0007
HPSE	4.88	0.0005
PLAU	4.71	0.0001
NOTCH4	4.34	0.0000
COL4A3	4.12	0.0045
EFNA1	3.66	0.0009
ITGB3	3.24	0.0002
CCL2	3.02	0.0002
JAG1	2.98	0.0003
TIMP1	2.10	0.0010
EGF	1.69	0.0509

PDGFA	-1.51	0.0046
THBS1	-2.03	0.0030
TIMP2	-5.35	0.0053
ID3	-13.12	0.0001
ID1	-46.63	0.0013

<u>Supplementary Table 2:</u> Genes significantly differentially expressed between 3D and 2D U87 GBM cultures.

Gene	Mean Fold Change 3D to 2D	P value (t-test)
CXCL9	143.01	0.00047
CXCL6	97.91	0.00067
CXCL5	87.63	0.00006
CXCL10	79.89	0.00109
ANGPT1	77.53	0.00055
HGF	77.35	0.00008
TEK	76.29	0.00160
EREG	75.93	0.00035
CXCL3	65.19	0.00032
PF4	60.97	0.00057
FGF1	53.69	0.00078
IFNA1	50.45	0.00040
IL1B	46.53	0.00246
CCL11	45.78	0.00026
FIGF	42.22	0.00265
ANGPTL3	39.67	0.00397
CDH5	38.41	0.00038
IGF1	38.23	0.00399
LEP	38.23	0.00092
TGFBR1	37.27	0.00325
COL4A3	36.25	0.00163

IFNG	34.86	0.00179
PLG	31.34	0.00016
CXCL1	28.77	0.00079
IFNB1	27.28	0.00010
ANPEP	26.72	0.00000
PROK2	26.42	0.00008
MMP9	24.03	0.00012
PLXDC1	23.97	0.00009
VEGFR1	22.63	0.00022
PTGS1	21.11	0.00146
TIMP3	20.97	0.00157
LECT1	19.29	0.00370
BAI1	18.34	0.00000
ANGPT2	16.56	0.00031
KDR	16.56	0.00020
HPSE	15.89	0.01141
VEGFC	12.24	0.00328
TNF	11.99	0.00041
ANGPTL4	11.26	0.00139
S1PR1	10.29	0.00293
STAB1	9.51	0.00280
THBS2	9.40	0.01247
SERPINF1	9.36	0.00920
IL6	9.25	0.00526
ITGAV	9.02	0.04540
CD31	8.17	0.00306

EGF	7.78	0.00118
IL8	7.29	0.00047
EFNA3	6.82	0.00158
CCL2	5.30	0.00959
TYMP	4.77	0.00002
HAND2	4.63	0.00576
TGFA	4.55	0.03053
EFNA1	3.43	0.01364
HIF1A	3.14	0.06359
FGF2	2.85	0.01056
NOTCH4	2.65	0.00216
MDK	1.95	0.03794
PDGFA	1.84	0.01370
TIMP1	-1.54	0.02214
FGFR3	-1.61	0.00851
NRP2	-1.63	0.02080
LAMA5	-1.73	0.00209
ITGB3	-2.20	0.00261
PGF	-2.41	0.04214
TGFB1	-2.50	0.00234
COL18A1	-3.06	0.00352
SPHK1	-3.48	0.03321
TNFAIP2	-3.82	0.00106
THBS1	-4.79	0.00102
JAG1	-5.38	0.00007
ID3	-5.94	0.00030

Supplementary Table 3: Full list of genes differentially expressed between a GBM primary tumor explant T7 / 11 cultured in the RCCS for 3 weeks and a monolayer culture derived from the T7 / 11 explant and cultured for six passages.

**Gene Symbol**	**T-TEST p value**	**Fold Up- or Down-Regulation Explant : T7/11 2D cell line**
MMP9	0.00546	633.265
CXCL3	0.00122	227.019
SERPINF1	0.00357	143.344
IL8	0.00070	73.347
STAB1	0.00134	64.000
IL6	0.00051	39.854
S1PR1	0.00752	32.074
IL1B	0.00310	30.274
ID1	0.03017	28.907
CXCL10	0.00412	22.264
PTGS1	0.00083	21.015
EFNB2	0.00257	20.773
CD31	0.00418	17.794
NRP2	0.00286	16.412
NOTCH4	0.00090	14.588
HPSE	0.00030	14.156
TNF	0.00149	13.548

KDR	0.00298	13.361
EFNA1	0.00819	11.158
HAND2	0.00415	8.938
TNFAIP2	0.01356	8.320
FGF1	0.00537	7.078
CXCL1	0.00344	5.952
TGFBR1	0.00149	5.789
CXCL5	0.00472	5.605
CXCL6	0.00338	4.627
MDK	0.04769	4.209
TGFA	0.00303	3.972
LECT1	0.02145	3.333
EGF	0.03119	3.102
ANGPTL3	0.01316	2.502
CDH5	0.02852	2.109
ITGAV	0.00511	2.037
FIGF	0.02784	1.849
PROK2	0.01963	-1.434
COL4A3	0.00042	-1.613
HPRT1	0.01101	-1.625
PF4	0.00029	-1.643
IFNG	0.00029	-1.643

PLG	0.00029	-1.643
IFNB1	0.00029	-1.643
TIMP3	0.00029	-1.643
EFNA3	0.00029	-1.643
CCL11	0.00577	-1.745
ID3	0.04226	-1.815
VEGFC	0.00067	-1.905
ANGPT2	0.00659	-2.033
PGF	0.01513	-2.178
TIMP2	0.00076	-2.901
HIF1A	0.00460	-3.074
PLXDC1	0.00023	-5.426
PDGFA	0.00069	-5.816
EREG	0.00204	-6.758
JAG1	0.00066	-8.897
THBS2	0.00001	-11.107
ANGPT1	0.00040	-14.254
THBS1	0.00001	-29.582
COL18A1	0.00005	-30.696

Supplementary Table 4: Genes significantly differentially expressed between HBMEC / KNS42 RCCS co-culture and KNS42 RCCS culture.

Gene	T-test p value	Fold Up- or Down-Regulation HBMEC KNS42 / KNS42
TEK	0.001497	-530.67
TIMP3	0.000126	-498.57
HGF	0.000200	-458.78
S1PR1	0.000272	-457.72
COL4A3	0.001531	-400.32
EGF	0.002181	-350.11
LEP	0.000395	-295.09
ANPEP	0.000037	-279.17
CCL11	0.000468	-262.29
STAB1	0.000057	-246.42
CXCL9	0.000238	-238.58
IFNA1	0.000457	-220.05
PLXDC1	0.000283	-211.57
HAND2	0.000053	-209.62
IFNG	0.000147	-203.89
VEGFR1	0.000563	-203.42
KDR	0.000241	-201.09
PROK2	0.000072	-196.49
PLG	0.000383	-196.49
ANGPT1	0.000444	-173.04
CDH5	0.000578	-168.31
CXCL10	0.000512	-163.33

CXCL5	0.000622	-162.20
MMP2	0.001326	-150.64
CD31	0.000051	-143.18
LECT1	0.003060	-140.56
IFNB1	0.000681	-137.66
FIGF	0.000052	-129.34
PF4	0.000531	-114.96
FGF1	0.000037	-82.42
TGFB2	0.000137	-64.22
SERPINF1	0.000461	-52.16
CCL2	0.000032	-50.50
ANGPT2	0.000160	-48.67
FGFR3	0.000204	-42.67
NOTCH4	0.000007	-39.44
TGFBR1	0.000327	-37.06
IGF1	0.001986	-30.31
ANGPTL3	0.000050	-22.03
THBS2	0.000537	-16.81
SPHK1	0.000201	-16.55
PGF	0.000007	-15.58
MMP9	0.000251	-13.28
EFNB2	0.000129	-12.71
NRP2	0.000155	-10.79
HPSE	0.000304	-10.40
PDGFA	0.000001	-9.68
EFNA3	0.017744	-7.56

THBS1	0.000018	-7.24
EPHB4	0.001502	-6.83
IL1B	0.001798	-6.15
PTGS1	0.000192	-4.73
TIMP2	0.001354	-3.34
ITGAV	0.000627	-2.86
B2M	0.000534	-2.58
VEGFC	0.000407	-2.16
IL8	0.002520	-1.78
IL6	0.011001	-1.70
CXCL3	0.012173	-1.51
FGF2	0.029402	-1.48
ID3	0.018180	1.27
RPL13A	0.007627	1.52
HPRT1	0.012060	1.97
HIF1A	0.000438	1.97
TGFA	0.042842	2.01
EFNA1	0.046569	3.14
COL18A1	0.023907	4.01
EREG	0.000002	5.16
PLAU	0.000065	14.95
MDK	0.001385	15.51
CXCL1	0.000047	21.28
ID1	0.028397	30.38

<u>Supplementary Table 5:</u> Genes significantly downregulated in KNS42 aggregates in response to VEGF inhibition with the VEGF inhibitor CBO-P11 relative to untreated aggregates.

Gene	P value (t-test)	Fold change VEGF treated/Untreated
TIMP3	0.000126	-332.37
HGF	0.000202	-240.52
LEP	0.000398	-196.72
PLXDC1	0.000283	-193.56
TEK	0.001522	-182.28
CCL11	0.000471	-174.85
CXCL9	0.000240	-159.05
STAB1	0.000057	-141.37
MMP2	0.001325	-141.04
VEGFR1	0.000568	-136.87
KDR	0.000243	-134.05
PROK2	0.000072	-130.99
PLG	0.000387	-130.99
EPHB4	0.000021	-123.07
ANGPTL4	0.000066	-121.66
ANGPT1	0.000448	-115.36
MMP9	0.000189	-110.41

IGF1	0.001806	-109.90
CXCL5	0.000629	-108.13
TNF	0.000495	-94.79
IFNB1	0.000690	-91.77
IFNG	0.000156	-86.82
EFNA3	0.005153	-84.25
CXCL6	0.000761	-82.71
PTGS1	0.000079	-76.82
PF4	0.000539	-76.64
JAG1	0.000007	-76.29
SPHK1	0.000012	-74.89
CD105	0.000791	-73.35
PLAU	0.000043	-69.87
LECT1	0.003163	-69.87
TGFA	0.000552	-64.00
ANGPTL3	0.000046	-62.97
ANPEP	0.000043	-61.53
BAI1	0.000204	-56.49
THBS2	0.000494	-54.57
IFNA1	0.000503	-53.20
ITGB3	0.000010	-48.39
CXCL10	0.000842	-44.94

VEGFA	0.003592	-42.91
TNFAIP2	0.000040	-36.50
TYMP	0.000004	-35.10
EREG	0.000121	-34.46
CDH5	0.000649	-34.46
FIGF	0.000064	-33.98
LAMA5	0.000002	-31.49
CD31	0.000073	-30.06
FGF1	0.000078	-28.77
HAND2	0.000089	-26.66
COL18A1	0.000680	-23.32
IL8	0.000131	-20.97
TGFBR1	0.000475	-19.97
IL1B	0.001157	-18.72
FGFR3	0.000245	-18.25
MDK	0.002540	-16.64
CXCL1	0.000260	-16.22
AKT1	0.000098	-12.91
IL6	0.000587	-12.04
ID3	0.000080	-11.52
SERPINF1	0.000926	-11.39
PGF	0.000007	-10.58

NOTCH4	0.000336	-8.67
ANGPT2	0.001329	-8.32
COL4A3	0.007616	-7.34
TGFB1	0.014281	-6.02
CXCL3	0.000763	-5.84
VEGFC	0.001310	-5.54
TGFB2	0.007900	-5.38
EFNA1	0.008076	-4.26
NRP1	0.008571	-4.13
EGF	0.048570	-4.11
NRP2	0.011570	-4.06
S1PR1	0.006910	-3.92
FGF2	0.022892	-3.60
CCL2	0.024887	-2.54

<u>Supplementary Table 6</u>: Genes significantly downregulated in KNS42 aggregates in response to FGF inhibition with the FGF receptor tyrosine kinase inhibitor PD166866 relative to untreated aggregates.

Gene	P value (t-test)	Fold change FGF treated /Untreated
TIMP3	0.000126	-411.10
HGF	0.000201	-375.67
TEK	0.001506	-300.94
LEP	0.000396	-243.31
PLXDC1	0.000282	-239.41
IL6	0.000360	-226.49
CCL11	0.000469	-216.27
STAB1	0.000057	-203.19
CXCL9	0.000239	-196.72
MMP9	0.000186	-189.14
MMP2	0.001318	-174.45
ANPEP	0.000038	-171.65
VEGFR1	0.000565	-169.29
IFNG	0.000147	-168.12
KDR	0.000242	-165.80
PLG	0.000384	-162.02
ANGPT1	0.000446	-142.68

CDH5	0.000581	-138.78
CXCL6	0.000741	-136.24
IGF1	0.001795	-135.92
CXCL5	0.000624	-133.74
EFNA3	0.005070	-127.70
SPHK1	0.000012	-124.50
CXCL10	0.000516	-123.35
TGFA	0.000533	-117.78
LECT1	0.003075	-115.89
IFNB1	0.000684	-113.51
CD31	0.000051	-111.95
TNF	0.000492	-111.43
EPHB4	0.000023	-107.14
JAG1	0.000007	-100.43
ANGPTL4	0.000066	-95.23
PTGS1	0.000078	-95.01
PF4	0.000534	-94.79
PROK2	0.000077	-92.84
CD105	0.000783	-90.72
IFNA1	0.000471	-85.04
ANGPTL3	0.000043	-79.16
ITGB3	0.000010	-75.06

THBS2	0.000448	-72.84
BAI1	0.000201	-69.87
PLAU	0.000041	-69.39
FIGF	0.000054	-65.34
ANGPT2	0.000154	-62.39
EREG	0.000205	-60.55
ACTB	0.009855	-58.76
SERPINF1	0.000454	-52.71
IL1B	0.001566	-45.89
TNFAIP2	0.000039	-45.15
TYMP	0.000003	-43.41
HAND2	0.000059	-37.97
TGFBR1	0.000330	-33.36
LAMA5	0.000001	-32.67
FGF1	0.000058	-31.20
IL8	0.003057	-27.47
FGFR3	0.000217	-25.75
CXCL1	0.000240	-24.70
MDK	0.001991	-21.36
COL18A1	0.000613	-20.68
VEGFA	0.004169	-19.70
NOTCH4	0.000009	-17.92

VEGFC	0.000033	-13.67
PGF	0.000006	-13.09
AKT1	0.000070	-12.24
COL4A3	0.002128	-12.21
CXCL3	0.000862	-11.16
CCL2	0.001480	-7.59
TGFB2	0.000242	-7.38
TGFB1	0.000365	-7.19
S1PR1	0.000728	-7.13
ID3	0.000307	-7.08
GAPDH	0.001512	-6.39
NRP2	0.000172	-5.66
EFNA1	0.001015	-5.64
RPL13A	0.001966	-4.97
EGF	0.005661	-4.89
NRP1	0.001125	-4.71
THBS1	0.002169	-4.67
FGF2	0.000106	-4.18
EFNB2	0.000675	-3.56
HPRT1	0.021663	-3.39
PDGFA	0.000384	-3.36
HIF1A	0.000113	-2.68

TIMP1	0.004945	-2.28
ITGAV	0.013000	-1.66

Supplementary Table 7: Genes significantly differentially expressed between U87 aggregates treated with the VEGF inhibitor CBO-P11 and untreated aggregates.

Gene	T- test p value	Fold change U87 aggregates VEGF treated : Untreated
CXCL9	0.000468	-257.48
CXCL6	0.000657	-178.73
CXCL5	0.000056	-156.32
TEK	0.001567	-127.26
PF4	0.000558	-111.30
CCL11	0.000251	-83.58
LEP	0.000884	-69.79
IGF1	0.003818	-69.79
IFNG	0.001703	-63.63
PLG	0.000154	-57.22
ANPEP	0.000001	-53.76
EREG	0.000360	-50.86
PROK2	0.000076	-48.22
VEGFR1	0.000199	-41.31
PTGS1	0.001339	-38.54
TIMP3	0.001448	-38.28
PLXDC1	0.000094	-35.88
LECT1	0.003394	-35.22

COL4A3	0.001669	-34.50
BAI1	0.000003	-33.47
STAB1	0.001811	-32.11
HGF	0.000098	-26.08
IFNA1	0.000434	-26.08
MMP9	0.000137	-16.93
CD31	0.001593	-15.08
IFNB1	0.000140	-15.05
HAND2	0.004711	-12.08
SERPINF1	0.011853	-7.54
TNF	0.000899	-7.32
FIGF	0.004590	-6.61
ANGPTL3	0.008695	-5.12
ANGPT1	0.001543	-4.68
KDR	0.002465	-4.59
TYMP	0.000149	-4.02
TGFBR1	0.015786	-3.67
EFNA3	0.005978	-3.46
FGFR3	0.012908	-3.06
FGF1	0.005092	-2.98
CDH5	0.009445	-2.76
NOTCH4	0.002631	-2.19

LAMA5	0.029174	-1.61
EGF	0.031802	-1.47
CXCL10	0.025892	1.47
TIMP1	0.015468	1.65
THBS2	0.029741	2.00
FGF2	0.011823	2.59
CCL2	0.046516	2.61
HPRT1	0.007114	3.34
HIF1A	0.002956	3.90
B2M	0.000197	4.51
MDK	0.001321	4.77
VEGFC	0.018316	4.82
TNFAIP2	0.000078	5.25
IL8	0.045095	10.09

<u>Supplementary Table 8:</u> Genes significantly differentially expressed between U87 aggregates treated with the FGFR inhibitor PD166866 and untreated aggregates.

Gene	T-test p value	Fold change U87 aggregates FGF inhibitor : Untreated
CXCL6	0.000664	-134.05
CXCL5	0.000057	-116.16
PF4	0.000559	-106.64
CXCL9	0.000480	-102.06
TEK	0.001639	-73.35
CCL11	0.000256	-68.12
IFNG	0.001707	-60.97
LEP	0.000898	-58.22
PLG	0.000155	-54.82
IGF1	0.003926	-52.35
EREG	0.000414	-44.53
TIMP3	0.001455	-36.67
COL4A3	0.001911	-34.78
LECT1	0.003449	-31.41
PTGS1	0.001399	-30.77
VEGFR1	0.000217	-30.70
PROK2	0.000114	-29.72
PLXDC1	0.000125	-28.12

BAI1	0.000005	-26.72
ANPEP	0.000026	-23.97
HGF	0.000165	-18.17
IFNA1	0.000620	-15.17
STAB1	0.007079	-15.07
MMP9	0.000457	-13.58
CD31	0.002812	-11.90
HAND2	0.003938	-10.53
IFNB1	0.000182	-9.83
TNF	0.021794	-7.45
KDR	0.007834	-5.70
TGFBR1	0.020469	-4.70
SERPINF1	0.027681	-4.66
FIGF	0.015243	-4.63
ANGPTL3	0.031742	-4.26
EFNA3	0.040365	-3.61
ANGPT1	0.012551	-3.25
TYMP	0.012601	-2.26
CXCL10	0.005741	2.93
